# Graptolites from glacial erratics of the Laerheide area, northern Germany

**DOI:** 10.1007/s12542-017-0345-9

**Published:** 2017-05-03

**Authors:** Jörg Maletz, Heinrich Schöning

**Affiliations:** 1 Department of Geological Sciences, FU Berlin, Malteserstrasse 74-100, 12249 Berlin, Germany; 2 Am Spielplatz 3, 34613 Schwalmstadt, Germany

**Keywords:** Glacial erratics, Moraines, Kame, Drenthe-Stadium, Germany, Palaeozoic, Graptolites, Geschiebe, Moränen, Kame, Drenthe-Stadium, Deutschland, Paläozoikum, Graptolithen

## Abstract

Ordovician and Silurian glacial erratics of the Laerheide area (Lower Saxony, north-western Germany) bear well-preserved graptolites. The faunas provide important information on the origin and transport direction of the sediments preserved in a kame, representing the Drenthe stadial of the Saalian glaciation. The faunas even include species not commonly encountered in the successions of mainland Sweden, from where the erratics presumably originated. The most common graptolites are from Upper Ordovician (Sandbian to Katian) limestones and from Katian black shales. More common, however, are greenish limestones, sand- and siltstones, often combined in the term ‘Grünlich-Graues Graptolithengestein’, in which upper Wenlock to Ludlow (upper Silurian) graptolites are common.

## Kurzfassung

Gut erhaltene Graptolithen können in ordovizischen und silurischen Geschieben aus der Laerheide (Niedersachsen) gefunden werden. Sie bieten Informationen zu Ursprung und Transportrichtung der Sedimente, die heute in einer Kamestruktur des Drenthe-Stadiums der Saale-Vereisung erhalten sind. Manche dieser Formen sind auch in den skandinavischen Abfolgen, aus denen das Material vermutlich stammt, nur selten zu finden. Die Graptolithen finden sich in Kalken des Oberen Ordoviziums (Sandbium–Katium) und Schwarzschiefern des Katiums. Häufiger sind jedoch die kalkigen oder siltig-sandigen Geschiebe des ‘Grünlich-Grauen Graptolithengesteines’ mit Graptolithen des Oberen Wenlock bis Ludlow (Oberes Silur).

## Introduction

Fossil-bearing glacial erratics have long been used to document transport and flow directions of glacial ice sheets from the place of origin of the material (e.g. Roemer 1862, 1885), even though modern methods like satellite imaging are now superseding this seemingly ‘old-fashioned’ method (e.g. Boulton et al. 2001). ‘Leitgeschiebe’ are still regarded as important tracers for the origins of North German glacial deposits (see Ehlers 2011) and include both sedimentary and crystalline rocks. The investigation of sedimentary erratics has been dominated by the collection and description of the fossils. These identify unambiguously the age of the sedimentary rocks and their origin if the sedimentary rock types are still exposed in the area of their origin. Spectacular fossils such as *Xenusion auerswaldae* Pompeckj, 1927 (probably originating from the Lower Cambrian Kalmarsund Sandstone of southern Sweden: Jaeger and Martinsson 1967; Hauschke and Kretschmer 2015) have been found in glacial erratics, and new taxa are even now being described from glacial(?) erratics originating from the Palaeozoic succession of Scandinavia (e.g. Botting and Rhebergen 2011).

**Fig. 1:**
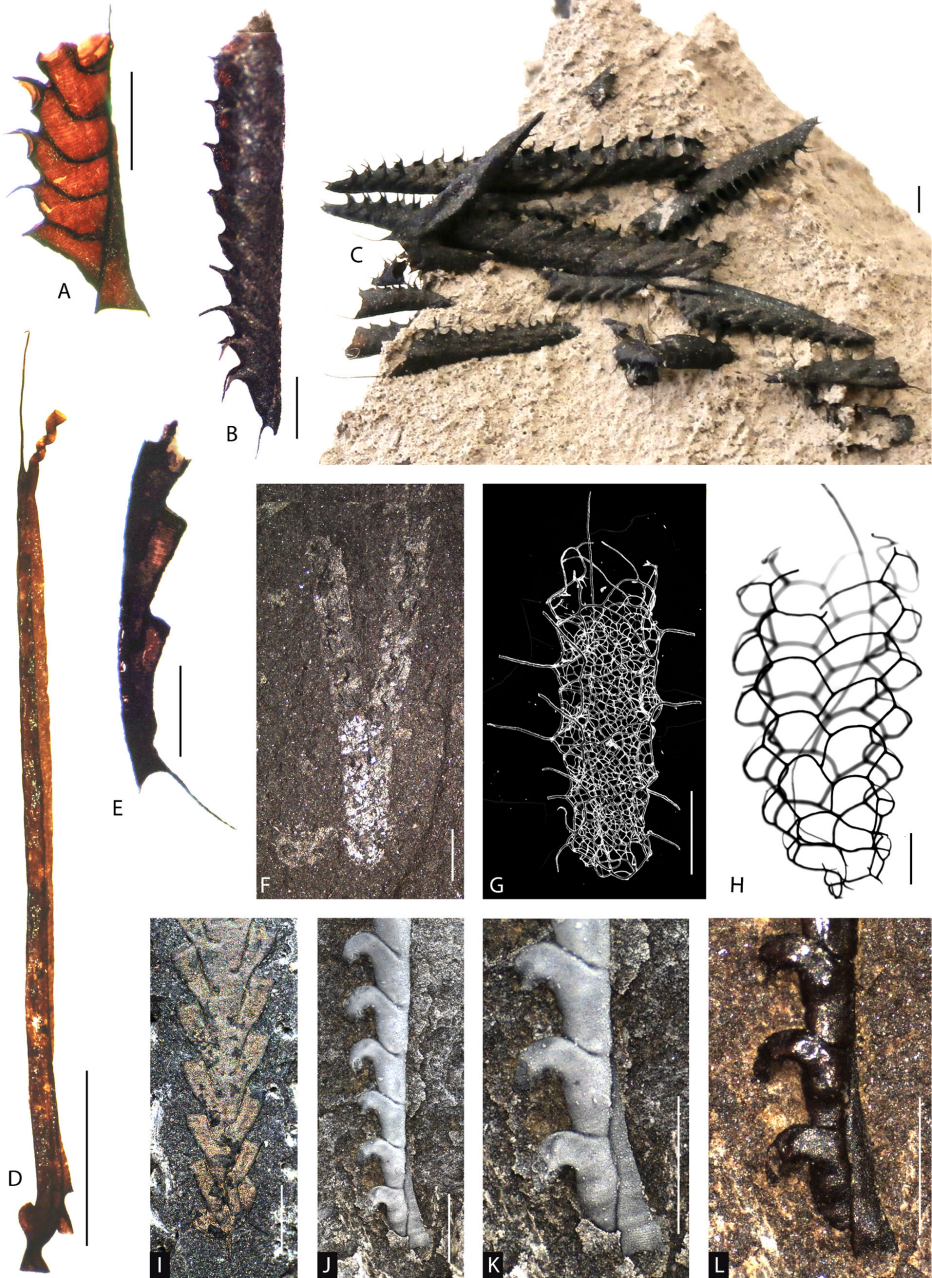
Graptolites from North German glacial erratics. **a** *Saetograptus leintwardinensis* (Hopkinson in Lapworth, 1880), SMF 68294, Nienhagen bei Rostock, Ludlow, Silurian. **b**, **c** *Saetograptus chimaera* (Barrande, 1850), SMF 75783, SMF 75784, Nienhagen bei Rostock, Ludlow, Silurian. **d** *Corynites wyszogrodensis* (Kozłowski, 1956), SMF 75785, Berlin, coll. Kühne, Katian, Ordovician. **e** *Bohemograptus* sp., proximal end, SMF 75786, B9/96, Bramsche, Mecklenburg-Vorpommern, Ludlow, Silurian. **f** *Dicranograptus clingani* (Carruthers, 1868), SMF 75787, Slg. Weil Nr. 18, Kieler Bucht, Katian, Ordovician. **g** *Spinograptus spinosus* (Wood, 1900), MB.G. 1078, Hiddensee, Rügen, scanning electron microscope (SEM) photo, Ludlow, Silurian. **h** *Plectograptus toernquisti* (Bates et al., 2006), SMF 75496, Bramsche, Mecklenburg-Vorpommern, Ludlow, Silurian. **i** *Rectograptus gracilis* (Roemer, 1861), PMU 31641, Röstånga, Scania, Sweden, partial relief specimen filled with pyrite, obverse view (not from glacial erratic), Katian, Ordovician. **j**–**l** *Monograptus priodon* (Bronn, 1835), SMF 75780, Kiesgrube Basedow, Mecklenburg-Vorpommern, coll. R. Klafack, **j**, **k** coated with ammonium chloride to better show the details, note preservation of fuselli in (**k**), Wenlock, Silurian. *Scale bars* represent 1 mm in all photos

Graptolites are relatively common and highly diverse in glacial erratics (Fig. 1). The earliest illustration of a glacial erratic block with graptolites may be by Walch (1771, suppl. IVc, Fig. 5) from Stargard, Mecklenburg. It took nearly a century, however, before Heidenhain (1869) and Haupt (1878) described these faunas in more detail. Gümbel (1878) may have been the first to isolate graptolites chemically from their calcitic matrix based on this kind of material. A great time for graptolite research started with Wiman (1895), who described in detail the construction of graptolite tubaria (an replacement term for rhabdosomes: Mitchell et al. 2013, p. 34) based on chemically isolated graptolites from glacial erratics, but also from samples collected in situ from a number of Scandinavian localities. One of the most detailed descriptions of chemically isolated graptolites remains Kraft’s (1926) investigation of *Rectograptus gracilis*, but the important works of Kozłowski (1938, 1949) on Tremadocian benthic taxa and Urbanek (1958) of Silurian monograptids are also highlights of graptolite research. There is no doubt that the study of Silurian retiolitids (e.g. Münch 1931; Eisenack 1935; Kozłowska-Dawidziuk 1995; Maletz 2008, 2010) would not have been possible without chemically isolated material, including that from glacial erratics (Fig. 1g, h).

Kowalski (1964, 1966a, b, 1968a, b, 1969, 1984) provided overviews on the graptolites found in glacial erratics in a number of short review papers. These show the most important taxa encountered, but unfortunately, the illustrated material is often not specimens of glacial origin, but based on available published illustrations from various sources, and therefore may be misleading. It appears that many of the taxa listed in various publications as originating from North German glacial erratics have never been properly described or illustrated from these erratics. Available descriptions and illustrations are not sufficient for modern identification (e.g. Heidenhain 1869; Haupt 1878; Roemer 1885), and the specimens are unfortunately not available for re-identification.

**Fig. 2:**
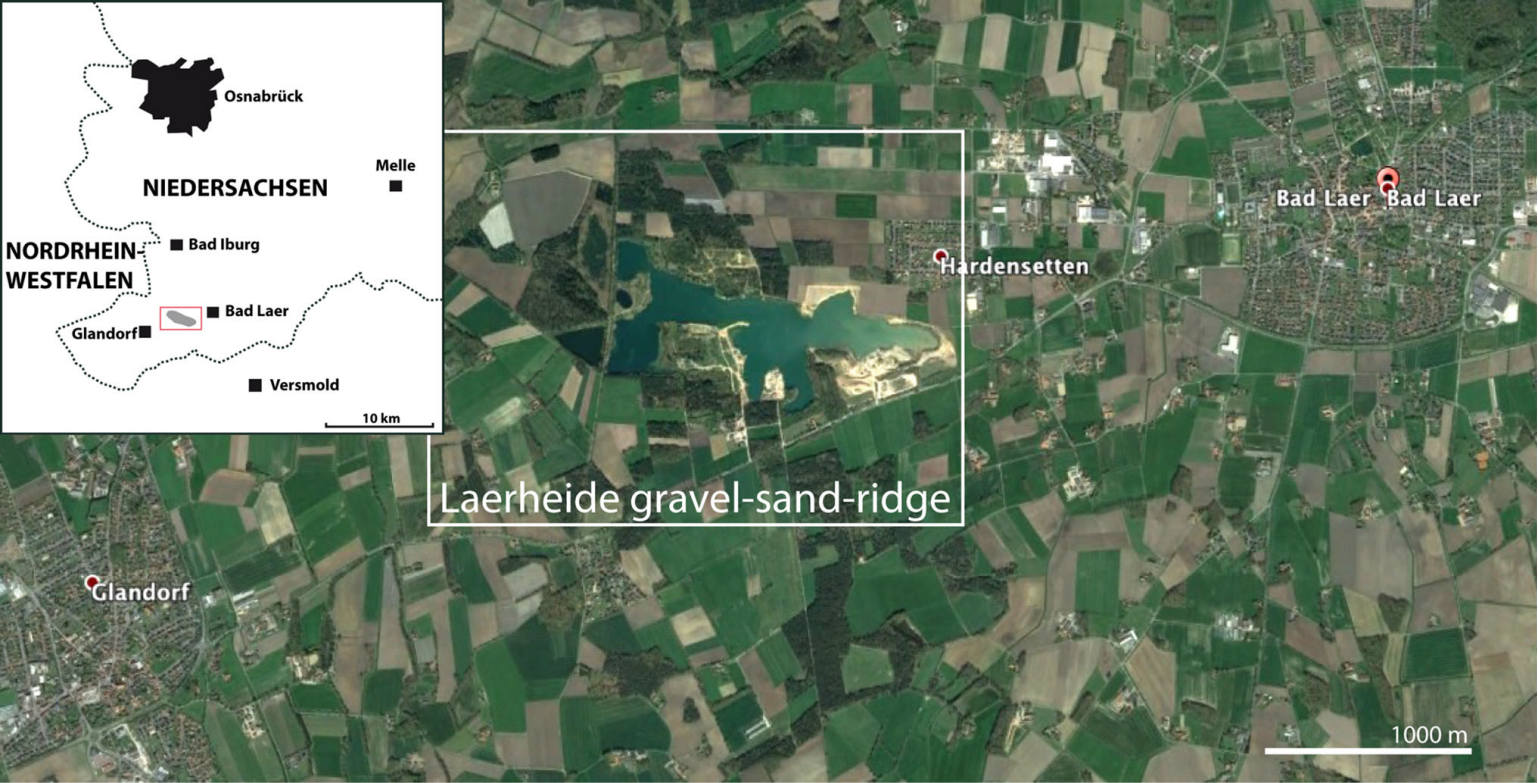
Map of the Laerheide locality (Google Earth).* Inset*: overview map based on Schöning (2002, Fig. 1)

Glacial erratics with Ordovician graptolites are either rarely encountered or have not attracted much attention, even though they may also bear excellently preserved faunas and even provide information on taxa not known from other sources. This is shown by the descriptions of *Archiretiolites regimontanus* by Eisenack (1935) and *Corynites wyszogrodensis* by Kozłowski (1956) (Fig. 1d), species that have been found only once or twice and are unknown from successions in situ. Other species have been described from Scandinavian successions, but important details were revealed only by isolated material, for example of *Gymnograptus linnarssoni* from western Pomerania, Poland (Urbanek 1959). Ordovician graptolites may be more common in glacial erratics than previously indicated (Fig. 2).

Ordovician graptolites from glacial erratics provide important constructional information due to the excellent preservation as relief specimens. One of the reasons for the previously poor record may be the lack of interest in this fossil group, but also the difficulty for most fossil collectors to get answers to questions on taxonomy and to find the relevant literature. Despite this, graptolites can provide important information for dating glacially deposited sediments and for the origination of the fossil-bearing rocks (Kalbe and Maletz 2015, p. 5). Unfortunately, the record of graptolites in glacial erratics has been limited to identification and taxonomic description (e.g. Kraft 1926; Gothan 1934; Eisenack 1935; Urbanek 1959; Zessin et al. 1994), and further evaluation of the enclosing sediments and their (palaeo-)geographical origin has not been undertaken.

Research on graptolites from glacial erratics has concentrated mostly on the Silurian faunas (see Jaeger 1959, 1978, 1991), as the ‘Grünlich-Graues Graptolithengestein’ (cf. Roemer 1862, p. 608) became famous—at least in Germany—for the presence of three-dimensionally and well-preserved graptolites (Fig. 1a–e). It is also the most commonly encountered graptolitiferous rock type in glacial erratics of Scandinavian origin. Jaeger (1991) and Maletz (2008) indicated that the time interval covered by this lithology ranges from the Homerian (Wenlock) *Cyrtograptus lundgreni* Biozone to the Ludfordian (Ludlow) *Bohemograptus cornutus*/*praecornutus* Biozone. Quite a number of Silurian monograptids have been described—in some cases exclusively—from glacial erratics (e.g. Jaekel 1889; Kühne 1955; Urbanek 1958; Jaeger 1991). In particular, glacial erratics from northern Germany have yielded excellently preserved material of a number of monograptids (Münch 1938; Kühne 1955; Jaeger 1959, 1991; Maletz 1999). Münch (1938) was one of the first to describe the proximal development of monograptids from chemically isolated material, and Eisenack (1942) named the sinus and lacuna stages of this development based on specimens he identified as *Monograptus* (now *Pristiograptus*) *frequens* Jaekel, 1889.

## The Laerheide locality

A characteristic gravel-sand ridge in the southern part of the ‘Osnabrück Landkreis’ WSW of Bad Laer in the Laerheide area (northern part TK 3841, Bl. Iburg, southern part TK 3914 Bl. Versmold) is the origin of the graptolite faunas documented here. The structure, lying about 7 km south of the southern ridge of the Teutoburger Wald, was originally ca. 2.5 km long and 600 m wide, but the remains are nearly completely gone (Schöning 1991). Keller (1951, p. 353) and Staude (1992) interpreted the ridge as a fluvio-glacial kame structure formed during the final stage of the Drenthe stadial of the Saale glaciation. Schöning (1991) discussed a NW–SE flow direction of the meltwaters depositing these sediments and recognized a fairly complex development of the structure, including folding and faulting of the sediments, partly through the action of the meltwater and the advancing and retreating Saalian ice shield.

Ehlers et al. (2011) and Böse et al. (2012) discussed the Saale glaciation, which is generally differentiated into the Drenthe and Warthe advances. The Dutch Drenthe and the Polish Odra glaciations are recognized as equivalent to the older Saalian ice advance of northern Germany, during which the ice sheet covered nearly all of Schleswig–Holstein and Niedersachsen and reached the southern margin of the Münsterland Bight. Tills in the upper part of the Saalian till unit are characterized by east Baltic erratics, largely Palaeozoic limestones, indicating a change in ice-movement direction from NNE-SSW to ENE-WSW towards the end of the glaciation (Ehlers et al. 2011, p. 153). Winsemann et al. (2016) referred the late Saalian glaciation to the MIS 6 and discussed glacial outburst flows in the Münsterland basin.

According to Skupin et al. (1993, p. 107), the Münsterland Bight was covered by four separate glacial advances during the Drenthe stadial. The Laerheide area was already covered through the first and largest advance of the glaciation, indicated by the dominance of southern Swedish crystalline erratics or ‘Leitgeschiebe’ (Zandstra 1993, p. 140). Younger ice advances with a higher proportion of east Fennoscandian erratics did not reach the area of the southern slope of the Teutoburger Wald (Skupin et al. 1993, p. 109). The sedimentary clasts within this glacial deposit largely comprise Palaeozoic rocks of Scandinavian origin and Upper Cretaceous rocks from the Baltic Sea region. The Plänerkalk from the Teutoburger Wald and sediments from the Osnabrücker Bergland represent sediment types of local origin (see Keller 1951; Schöning 1977, 2000). Among the Palaeozoic sedimentary rocks of Scandinavian origin are mainly Ordovician limestones of a variety of lithologies: red, grey and black ‘Orthoceratite limestones’ of Middle Ordovician age and various limestones of Late Ordovician age.

The Palaeozoic glacial erratics of the Laerheide area bear a diverse fauna of Middle Cambrian to upper Silurian trilobites (Schöning 1978, 1982, 1986, 1996, 2002, 2010, 2014; Schöning and Popp 2015), and also phosphatic and calcitic brachiopods, gastropods (Amler et al. 2002), ostracods (Schallreuter 1996; Schallreuter and Hinz-Schallreuter 2005) and corals. Graptolites are uncommon, but a number of taxa have been discovered, mostly as individual specimens or fragments. The Silurian blocks often include richer assemblages than the Ordovician ones. The graptolite-bearing glacial erratics are mainly light-coloured limestones, and the specimens are preserved in partial to full relief. A few slabs of hard black shale with Upper Ordovician graptolites may be referred to the Middle *Dicellograptus* Shale (cf. Hadding 1915; Pålsson 2001) of Late Ordovician age. About a dozen limestone and shale boulders with Silurian graptolites have also been found, bearing mainly Ludlow monograptids and a few fragments of retiolitids.

It is interesting that early Silurian (Llandovery) erratics appear to be lacking in the region—at least graptolites of this time interval have not been discovered. Llandovery successions with graptolites are present, however, in southern Scandinavia. The investigation of glacial erratics with trilobites shows the same story. Very few Llandovery trilobites have been found in the limestones, in contrast to the much higher number of Ordovician and higher Silurian taxa. A possible explanation is that the southern Scandinavian successions include a high amount of soft shales in the Lower Silurian (see Bjerreskov 1975: Bornholm). These may have been destroyed through the long transport, while the harder and more resistant limestones and silt- to sandstones of the Ludlow survived (e.g. the limestones of the Grünlich-graues Graptolithengestein). Most of the graptolites from the Laerheide locality are found in limestones, even though some Upper Ordovician taxa may be found in hard, possibly silicified black mudstones.

## Ordovician graptolites

**Fig. 3:**
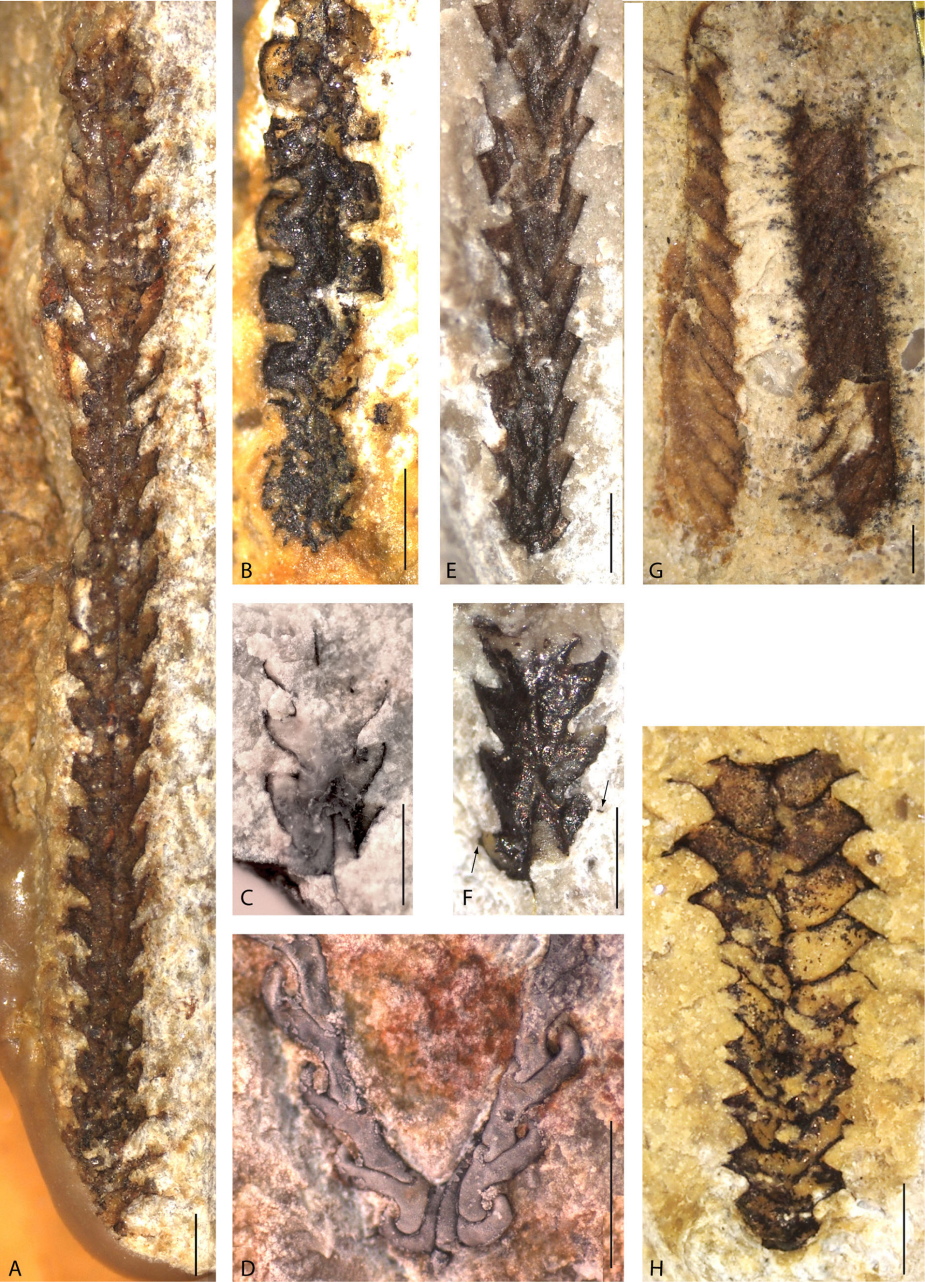
Ordovician graptolites from the Laerheide. **a** *Oepikograptus bekkeri* (Öpik, 1927), SMF 75788, proximal end slightly incomplete. **b** Climacograptid indet, SMF 75789, showing thecal outlines and zigzag median septum, proximal end incomplete. **c**, **f** *Hustedograptus* sp., SMF 75781b, SMF 75781c, two proximal ends in obverse view. **d** *Jiangxigraptus vagus* (Hadding, 1913), SMF 75781a, proximal end of specimen with slightly longer stipes, coated with ammonium chloride. **e** *Rectograptus gracilis* (Roemer, 1861), SMF 75790, proximal end in reverse view, nearly flattened. **g** *Didymograptus* sp. of *D. murchisoni* type, SMF 75791, proximal end missing. **h** *Gymnograptus ejuncidus* (Berry, 1964), SMF 75792, proximal end in reverse view. *Scale bars* represent 1 mm in all photos

Quite a few Ordovician limestones provided well-preserved graptolites (Fig. 3). Most graptolites can be referred to the Axonophora, the most common group in the Middle to Upper Ordovician. For most taxa, only a single specimen is available from this investigation. Thus, it is not possible to determine faunal diversity and composition. The faunal elements indicate a Middle to Upper Ordovician (Darriwilian to Katian) age.

A number of hard black shale slabs bear poorly preserved biserial graptolites that may be referred to the Middle *Dicellograptus* Shale of Bornholm, Denmark and Scania, southern Sweden. The material is too poorly preserved to be identified and is not illustrated herein. Interestingly, specimens of *Dicranograptus clingani* (Fig. 1f) from this time interval have been described more than once from other localities bearing glacial erratics in northern Germany (Kowalski 1966a; Maletz 1995a) and provide some indication of the age and origin of the material. The species has not been found, however, in the Laerheide area. Generally, these graptolites are preserved as a flattened film of black organic material on black shales. The organic material of the graptolites often has a slight silvery sheen due to subsequent burial and heating of the sediments, and may be covered by pressure shadow minerals (see Maletz and Steiner 2015), but often shows little contrast with the sediment.

### *Didymograptus* sp. (Fig. 3g)

The specimen shows two wide distal stipes with the thecal apertures facing each other, but the proximal end is lacking. Thus, it can only be identified as *Didymograptus* sp. Specimens of the genus *Didymograptus* are characteristic for the middle part of the Darriwilian, and are restricted to the high-latitude regions (cold water regions) of the Ordovician (Goldman et al. 2013). They can be found typically in the black shales of the Elnes Formation of Norway and the corresponding Almelund Shale of the mainland of Sweden (Maletz 1995b, 1997b). Specimens may also be found in the Darriwilian limestones of Öland, but graptolites are rare in these, and specimens have been recorded only once. Bulman (1936) illustrated a well-preserved specimen of the slender *Didymograptus spinulosus* under the name *Didymograptus minutus* from Hälludden, Öland. Jaanusson (1960) described chemically isolated material of *Didymograptus* cf. *murchisoni murchisoni* from the Seby Limestone of Öland, associated with *Pseudamplexograptus distichus* and *Didymograptus pakrianus* (=*D. murchisoni*, mature specimens with proximal overgrowth) from the Ordovician of Estonia.

### *Oepikograptus bekkeri* (Fig. 3a)

A single, proximally incomplete specimen can be identified as *Oepikograptus bekkeri* (Öpik, 1927). The species is very characteristic, bearing a number of spined proximal thecae. It appears to be an extremely rare species and has been found in only a few Baltic localities (cf. Öpik 1927; Bulman 1932 (as *Climacograptus haljalensis*); Strachan 1959; Mitchell 1987). Öpik (1927) isolated the species from limestones, but stated that his material was not found in situ. He assumed that the material originated from rocks of the Kukruse Stage (Lower Sandbian: Cooper and Sadler 2012). Strachan (1959) reported the species from the boulders of the Tvären impact (Lindström et al. 1992, 1994), ca. 70 km N of Stockholm. Goldman et al. (2015) described and illustrated the species from the Kandava-25 drill core of Latvia, where the species occurs in an interval tentatively referred to the upper *Nemagraptus gracilis* or lower *Climacograptus bicornis* Biozone. The record in a glacial erratic from northern Germany is the first find in material of glacial origin and outside of Scandinavia.

### *Climacograptids* indet. (e.g. Fig. 3b)

A number of poorly preserved specimens can be identified as ‘climacograptids’ in the broadest sense, based on their strongly geniculate thecae. A precise identification is impossible, as important characters are not visible. The specimens show strongly geniculate thecae, with a convex ventral side of the thecae and a strong zig-zag median septum. They may be referred to *Haddingograptus*, *Pseudoclimacograptus* or even *Archiclimacograptus*, but due to the lack of detail of the proximal ends, a more precise identification is impossible.

### *Gymnograptus ejuncidus* (Fig. 3h)

A slender *Gymnograptus* specimen is here identified as *Gymnograptus ejuncidus* (Berry, 1964). The species is more slender than typical *G. linnarssoni* (Moberg, 1896), but the development of the apertural spines on the thecae is unknown. Urbanek (1959) described isolated material of *G. linnarssoni* and *Gymnograptus* sp. (=*Gymnograptus ejuncidus*) from glacial erratics found in Jaroslawiec on the Baltic Coast of western Pomerania, Poland. Hadding (1913) described *Gymnograptus* from the Lower *Dicellograptus* Shale of Scania. One of these specimens (Hadding 1913, pl. 3, Fig. 14) can be referred to *Gymnograptus ejuncidus*. *Gymnograptus* species are characteristic of the upper Darriwilian of low-latitude regions and appear first in the *Jiangxigraptus vagus* Biozone (previously the *Hustedograptus teretiusculus* Biozone) (see Maletz et al. 2007, 2011).

### *Jiangxigraptus vagus* (Fig. 3d)

A single dicellograptid is present in the collection, preserved in full relief in obverse view. The specimen shows the sicula to be attached along its complete length to stipe two. The specimen is here referred to *Jiangxigraptus vagus* (Hadding, 1913), following the identification of isolated material by Goldman et al. (2013). The genus *Dicellograptus* has been differentiated into a number of closely related genera in recent years (see Mu et al. 2002). As the type species of *Dicellograptus* bears a vertically positioned sicula, not attached to stipe 2, the genus *Jiangxigraptus* is used for species in which the sicula is partly or fully attached to stipe 2.

### *Hustedograptus* sp. (Fig. 3c, f)

Two small specimens of *Hustedograptus* sp. were found to be associated with *Jiangxigraptus vagus* on a single piece of limestone. The genus *Hustedograptus* is very long-ranging in the Middle to Upper Ordovician, and species are often difficult to identify. The specimens illustrated here show a sicula without antivirgellar spines, a distinctly asymmetrical proximal end, and simple, somewhat introverted thecal apertures. The details of the thecal apertures are uncertain, as lateral lappets cannot be recognized. Vague indications of apertural spines on the first thecal pair are visible in one specimen (Fig. 3f; arrows). Both specimens show the obverse view of a possible pattern A astogeny with a complete median septum.

### *Rectograptus gracilis* (Fig. 3e)

*Rectograptus gracilis* (Roemer, 1861) is a well-known species and commonly collected from glacial erratics in northern Germany. The species was recently illustrated from a local glacial erratic found on the island of Öland (Kalbe and Maletz 2015). It is typically found in the rock termed ‘Ostseekalk’ by German fossil collectors. Kraft (1926) first described it in all available detail from chemically isolated material. Bulman (1932) also described the species in great detail and mentioned its occurrence in loose blocks of the ‘Östersjökalk’ at Aarhus (Denmark) and Visby (Gotland, Sweden). He noted that the species was known only from loose blocks and that its stratigraphic age was somewhat uncertain. Related species are common in the Upper Ordovician worldwide. Goldman and Bergström (1997, p. 1002) suggested that many of the described species have ‘undoubtedly been erected on the basis of preservational differences, stratigraphical position and national origin’.

## Dendroid graptolites (Fig. 4j, l)

**Fig. 4:**
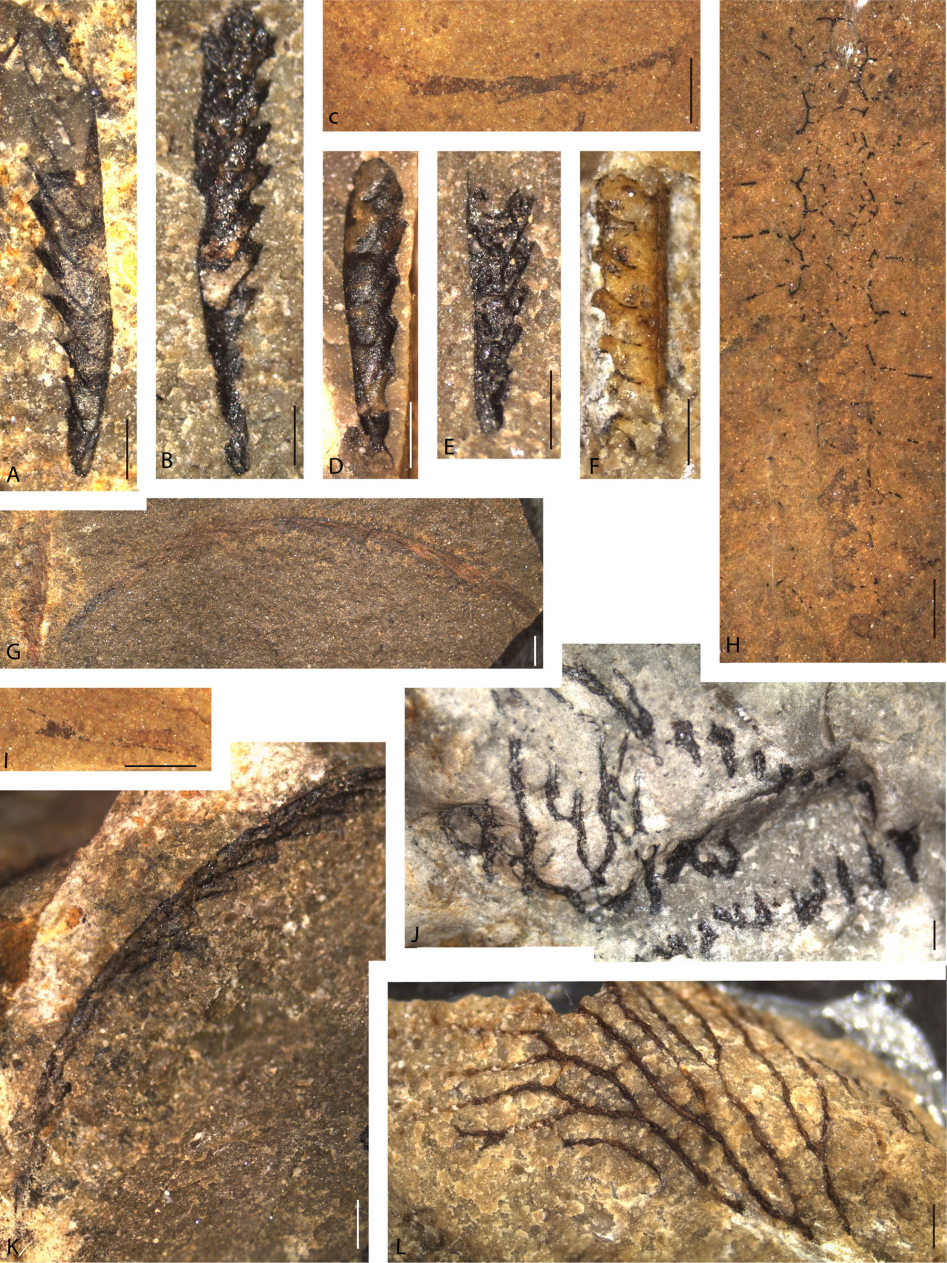
Silurian graptolites and Ordovician dendroid graptolites. **a**, **b** *Pristiograptus frequens* (Jaekel, 1889), SMF 75793, SMF 75794. **d**, **e** *Pseudomonoclimacis dalejensis* (Bouček, 1936), SMF 75795, SMF 75796. **f** *Monograptus flemingii* (Salter, 1852), SMF 75797, fragment in relief, sediment fill. **g**, **k** ?*Bohemograptus* sp., SMF 75798, SMF 75799, poorly preserved specimens without sicula. **h** *Spinograptus spinosus* (Wood, 1900), SMF 75800, poor and incomplete specimen. **c**, **i** *Neodiversograptus* sp.? SMF 75801. **j** Dendroid graptolite indet, SMF 75802. **l** ?*Dictyonema* sp., SMF 75803. *Scale bars* represent 1 mm in all photos

Dendroid graptolites appear to be uncommon in the glacial erratics in the Laerheide area, but a few fragments are available. None of these is identifiable to species level, as the colony shape and thecal details are not visible. One fragment shows indications of a larger colony and an irregular shape (Fig. 4j). The stipes are clearly arranged in a plane, but no thecal apertures are recognizable. The sediment is a grey limestone, identifiable as *Macrourus* Limestone of Sandbian to Katian (Late Ordovician) age, bearing *Toxochasmops macrourus* and brachiopod fragments.

A second specimen shows very slender stipes and a number of branching divisions (Fig. 4l), similar to species of the genus *Dictyonema*, but the fragment does not provide any information on the colony shape. Dissepiments, typical of the genus *Dictyonema*, are recognizable, even though poorly preserved. The sedimentary rock is a greyish-brown to light brown fine-grained limestone with the trilobites *Ascetopeltis* cf. *bockeliei*, *Panderia* sp., *Parillaenus* sp., *Remopleurides* sp., various ostracods, including *Platybolbina* sp., *Ectoprimitia corrugata* and *Bolbina* sp., indicating a Hirnantian (Late Ordovician) age.

## Silurian graptolites

The Silurian graptolite fauna (Fig. 4a–i, k) consists largely of monograptids, but poorly preserved retiolitids were also encountered. The faunal elements mostly indicate a Ludlow, late Silurian age, but a few Wenlock graptolites have also been found.

Radzevičius et al. (2010) described Silurian graptolites from glacial erratics from Mokrzeszów Quarry, Poland. The locality, at that time known as Nieder-Kunzendorf, Schlesien, was previously discussed by Roemer (1885), who investigated and illustrated the graptolite fauna. Jaekel (1889) also described and illustrated material from this locality. The material represents the southernmost point at which glacial erratics of the Saalian glaciation can be found.

### *Spinograptus spinosus* (Fig. 4h)

*Spinograptus spinosus* (Wood, 1900) is one of the common and easily recognizable retiolitid graptolites of the upper Silurian. It has been recovered from glacial erratics a number of times (see Maletz 2010), although it was originally described from shale material. The long lateral apertural spines and typical meshwork of the ancora sleeve are easily recognizable even in poorly preserved specimens (Fig. 4h). Chemically isolated material from glacial erratics can provide much better material of this species (Fig. 1g). The development of this species is considerably more complex than that of another typical Ludlow retiolitid genus, *Plectograptus*, here illustrated by *P. toernquisti* (Fig. 1h). Kowalski (1969, Fig. 29) also illustrated a specimen of this species, although the specimen is not from a glacial erratic, but is from Bykoš, Czech Republic (Bouček and Münch 1952, Fig. 10C).

### *Monograptus flemingii* (Fig. 4f)

The material from the Laerheide is quite poorly preserved and fragmentary, but based on the short interthecal septae and small proportion of the theca taken up by the thecal hook, it may be referred to *Monograptus flemingii* (Salter 1852). *Monograptus flemingii* is similar to *Monograptus priodon* (Bronn, 1835), which is much more common in glacial boulders (Fig. 1j–l). Urbanek (1958) described isolated material of *M. priodon* from glacial erratics from Poland. The material is fragmentary, but better preserved material from successions in Arctic Canada (Lenz 1974) shows details of the colony shape. Relief specimens of *M. priodon* are common in some glacial erratics and make the species one of the most attractive taxa to find (Fig. 1j–l). The illustrated material from Mecklenburg shows the species preserved in full relief. Even the fusellar rings on the sicula are recognizable at higher magnification when the specimen is coated with ammonium chlorite (Fig. 1k), but when uncoated, these details are not visible (Fig. 1l). *Monograptus priodon* was not found at Laerheide.

### *Saetograptus chimaera*

*Saetograptus chimaera* is present as a number of fragments, but no complete specimens with proximal end have been recognized, even though the species is the most commonly encountered monograptid in glacial erratics and has often been illustrated from these (e.g. Münch 1938; Walker 1953; Kühne 1955; Urbanek 1958). Thus, it is not illustrated from the Laerheide locality here. A typical example of a partly dissolved limestone showing the orientation of numerous specimens of *S. chimaera* (Fig. 1c) from the well-known locality Nienhagen in Mecklenburg shows the usual appearance of this species. The specimens are current aligned in this example and represent a monospecific assemblage. A completely isolated specimen from the same slab (Fig. 1b) shows the tubarium with the slender, straight sicula and the paired spines on the thecal apertures. Maletz (1996, 1997a) described a closely related species as *Saetograptus* sp. cf. *Saetograptus leintwardinensis* (Hopkinson in Lapworth, 1880) from a glacial erratic collected at Nienhagen, near Rostock. Štorch et al. (2014) recognized this material to be identical to *Saetograptus leintwardinensis* (Fig. 1a). The species has been found only once in a glacial erratic. It is closely related to *S. chimaera*, but can be separated through its typical trumpet-shaped sicula.

### *Pseudomonoclimacis dalejensis* (Fig. 4b, e) (=*Monograptus haupti* of Kühne 1955 and Jaeger 1991)

*Pseudomonoclimacis dalejensis* (Bouček, 1936) is a common but often misidentified species from the Ludfordian (upper Ludlow). It has been found a number of times in glacial erratics, and Kühne (1955) described it for the first time in some detail. Maletz (1999, Fig. 3) demonstrated the presence of sicular annuli in this species from chemically isolated and bleached material. Štorch et al. (2014) synonymized *Monograptus haupti* with the species *Pseudomonoclimacis dalejensis* from the Czech Republic.

### *Pristiograptus frequens* (Fig. 4a, d)

Pristiograptids of the *Pristiograptus dubius* (Suess 1851) type are among the most common monograptid species in North German glacial erratics and have frequently been described. They are often identified as *Pristiograptus dubius frequens* (Jaekel, 1889) or *Pristiograptus frequens* (Jaekel, 1889) and are present in most collections of chemically isolated material (Münch 1938, 1952; Jaeger 1991; Maletz 1999). Štorch et al. (2014) provided a modern taxonomic description of the species. The main problem with the taxon is the long biostratigraphic range of *Pristiograptus dubius* and the description of numerous closely related and difficult to separate species and subspecies (see Urbanek et al. 2012). The *P. dubius* group ranges at least from the Sheinwoodian (lower Wenlock) to the basal Pridoli (uppermost Silurian) without much change in the construction of the tubarium.

### *Bohemograptus* sp. indet. (Fig. 4g, k)

A few slender curved monograptid fragments are here identified as *Bohemograptus* sp. indet. They do not show any thecal details and are too poorly preserved for specific identification. *Bohemograptus* is a common genus in the Ludlow glacial erratics and has been described from chemically isolated material (Urbanek 1970). The proximal ends can easily be recognized through the curved sicula (Fig. 1e). Other slender fragments may be identified as *Neodiversograptus* sp. based on the simple thecal style and the slender proximal ends (Fig. 4c, i).
